# Captopril attenuates oxidative stress and neuroinflammation implicated in cisplatin-induced cognitive deficits in rats

**DOI:** 10.1007/s00213-024-06706-6

**Published:** 2025-01-15

**Authors:** Fatma Mostafa, Eman M. Mantawy, Riham S. Said, Samar S. Azab, Ebtehal El-Demerdash

**Affiliations:** 1 Department of Pharmacology and Toxicology, Faculty of Pharmacy, Ain Shams University, Cairo, Egypt; 2 Department of Drug Radiation Research, National Center for Radiation Research and Technology, Egyptian Atomic Energy Authority, Cairo, Egypt

**Keywords:** Cisplatin, Chemobrain, Captopril, Oxidative stress, Neuroinflammation

## Abstract

**Rationale:**

One of the most debilitating drawbacks of cisplatin chemotherapy is neurotoxicity which elicits memory impairment and cognitive dysfunction (chemobrain). This is primarily triggered by oxidative stress and inflammation. Captopril, an angiotensin-converting enzyme inhibitor, has been reported as a neuroprotective agent owing to its antioxidant and anti-inflammatory effects.

**Objective:**

We examined the possible neuroprotective effect of captopril against cisplatin-induced neurological and behavioral abnormalities in rats.

**Methods:**

Chemobrain was induced in rats by cisplatin (5 mg/kg, i.p.) on the 7th and 14th days of the study while captopril was administered orally (25 mg/kg) daily for three weeks. The effects of captopril were assessed by performing behavioral tests, histological examination, and evaluation of oxidative stress and inflammatory markers.

**Results:**

Cisplatin caused learning/memory dysfunction assessed by passive avoidance and Y-maze tests, decline in locomotion, and rotarod motor balance loss which were further verified by neurodegeneration observed in histological examination. Also, cisplatin aggravated oxidative stress by elevating lipid peroxidation (MDA) levels and diminishing catalase activity. Moreover, cisplatin upregulated the neuroinflammatory markers (TNF, IL-6, GFAP, and NF-κB). Captopril successfully ameliorated cisplatin damage on the levels of neurobehavioral and histopathological changes. Mechanistically, captopril significantly diminished MDA production and preserved catalase antioxidant activity. Captopril also counteracted neuroinflammation through inhibiting NF-κB and its downstream proinflammatory cytokines besides repressing astrocyte activity by reducing GFAP expression.

**Conclusion:**

Our findings revealed that captopril could abrogate cisplatin neurotoxicity *via* reducing oxidative stress and neuroinflammation thus enhancing cognitive and behavioral performance. This could suggest the repurposing of captopril as a neuroprotective agent, especially in hypertensive cancer patients receiving cisplatin.

## Introduction

Chemotherapy-related cognitive impairment is one of the devastating drawbacks for cancer patients treated with chemotherapy. It affects up to 75% of cancer patients during the treatment and may continue for several months post-treatment (Janelsins et al. 2014).These neurological complications have been encountered in patients with different malignancies such as colorectal, and gynecological malignancies, with a high incidence rate (17–75%) in women with breast cancer (Cerulla et al. 2019). Indeed, several chemotherapeutic agents have been reported to elicit cognitive impairment in cancer patients including doxorubicin, methotrexate, cisplatin, cyclophosphamide, 5-fluorouracil, and paclitaxel (Mounier et al. 2020; Jaiswara and Shukla 2023; Ibrahim et al. 2024). This cognitive dysfunction is manifested as short-term memory loss and deficits in attention, executive tasks, and speed of information handling which negatively impact the quality of life (QOL) of cancer patients (Országhová et al. 2021). This phenomenon is termed as chemobrain which prominently attracts the attention of researchers nowadays. Therefore, there is a growing concern for developing an effective strategy to improve the cognitive performance of surviving patients.

It is noteworthy that cisplatin (cis-diamminedichloroplatinum-II) represents one of the clinically efficient chemotherapeutic options for several malignancies like lung, colorectal, breast, bladder cancer, and brain tumors (Brown et al. 2019). Nevertheless, it triggers various threatening complications including nephrotoxicity, hepatotoxicity, neurotoxicity, and ototoxicity that lessen its clinical utility (Ghosh 2019). Indeed, cisplatin-induced neurotoxicity has been evidenced in several preclinical and clinical studies manifested chiefly as memory and cognitive deficits (Troy et al. 2000; Amidi et al. 2017; Mahmoud et al. 2023). Of note, the long-term behavioral changes are also observed in pediatric oncology patients with CNS and non-CNS malignancies. Besides, numerous in-vivo experiments have reported that newborn rats are more prone to cisplatin toxicities than adult ones (Piccolini et al. 2015; Fulco et al. 2018).

Although the mechanism of chemotherapy-induced cognitive impairment is still unclear, oxidative stress and neuroinflammation are considered the principal mechanisms behind this neurocognitive disorder (Gupta et al. 2022). Accumulating evidence has stated that excessive generation of reactive oxygen species (ROS) is triggered by cisplatin hence, provoking neuronal injury in the cortex and hippocampus which are the vital centers for learning and memory (Rendeiro et al. 2016; Das et al. 2020; Yoo et al. 2021). Moreover, the redox imbalance is tightly linked with neuroinflammation driven by microglial activation where ROS induces activation of multiple redox-responsive pro-neuroinflammatory mediators (Simpson and Oliver 2020). Amongst them, nuclear factor kappa B (NF-κB), which is considered the fundamental transcription factor governing the expression of a varied assortment of pro-inflammatory genes like tumor necrosis factor-alpha (TNF) and a diversity of interleukins (Lingappan 2018). Remarkably, the noxious ROS and pro-inflammatory biomolecules are considered the major neurotoxic stimuli that cooperate together in eliciting neuronal cell injury and consequently prompting behavioral and cognitive dysfunction associated with chemotherapy (Bagnall-Moreau et al. 2019).

It’s well known that hypertension is the most common comorbidity observed in cancer patients and that the majority of anticancer agents can exacerbate hypertension (Van Dorst et al. 2021). Moreover, hypertension can lead to further decline in cognitive functions (Walker et al. 2017). Captopril is an angiotensin-converting enzyme inhibitor (ACEI) that has been extensively utilized in the clinical practice for the management of hypertension in addition to a diversity of cardiovascular disorders including some forms of congestive heart failure (Messerli et al. 2018). Captopril is considered a safe and extensively utilized antihypertensive medication, however, skin rash, nausea, coughing, fatigue, headaches, and dizziness are frequently reported adverse effects associated with its administration (Takuathung et al. 2022). As a thiol-containing compound, captopril can effectively scavenge ROS, exert potent antioxidant properties, and eventually guard tissues from oxidative damage (Fouad et al. 2013; Karimani et al. 2018). Moreover, captopril has exhibited prominent anti-inflammatory effects through inhibiting NF-κB and subsequently repressing the transcription of various inflammatory genes (Gan et al. 2018; Mostafa et al. 2019). Due to the latter effects, captopril neuroprotective activity has been confirmed in several neurodegenerative diseases such as Parkinson’s disease (PD), and Alzheimer’s disease (AD) (Muñoz et al. 2006; Sonsalla et al. 2013; Abareshi et al. 2016; Ishola et al. 2022). Based on these valuable pharmacological activities, captopril can be considered a promising candidate to combat neurodegeneration and cognitive decline associated with chemotherapy. Therefore, the present study was intended for examining the cognitive improving effects of captopril using a formerly stated neonatal rat model of cisplatin-induced cognitive impairment (John et al. 2017; Owoeye et al. 2018). Additionally, this experiment also explored the possible mechanisms triggering the neuroprotective efficacy of captopril in an attempt to repurpose its use in cancer patients especially those suffering from hypertension.

## Materials & methods

### Drugs & reagents

Captopril was provided by SmithKline Beecham Co. Cairo, Egypt. Cisplatin was purchased as Cisplatine (Mylan, Italy). Malondialdehyde (MDA) content was chemically assayed based on the method of Mihara and Uchiyama (Uchiyama and Mihara 1978) and catalase activity kit was purchased from Biodiagnostics Co., (Giza, Egypt). Rat TNF ELISA kit was obtained from Fine Test, (Wuhan, China). A Rat IL-6 ELISA kit was obtained from SunLong Biotech, (HangZhou, China). The primary and secondary antibodies for immunohistochemistry were bought from (Thermo Fisher Scientific-Invitrogen, USA): Glial fibrillary acid protein (GFAP) mouse monoclonal primary antibody (Catalog # 14-9892-82; RRID: AB_10598206) and NF-κB rabbit polyclonal primary antibody (Catalog # PA5-27617; RRID: AB_2545093). Goat anti-mouse biotin secondary antibody (Catalog # 31800; RRID: AB_228305) and biotinylated goat anti-rabbit secondary antibody (Catalog # 65-6140; RRID. AB_2533969).

### Laboratory animals

The study procedures were conducted and accepted by the Institutional Animal Research Ethics Committee of the Faculty of Pharmacy, Ain Shams University, Cairo, Egypt (No #258). Female Sprague–Dawley rats (3 weeks old, weighing 40–50 g) were purchased from the Nile Co. for Pharmaceuticals and Chemical Industries (Cairo, Egypt). Rats were held in the animal facility of the Faculty of Pharmacy, Ain Shams University during the whole experiment. The rats were maintained in a regulated atmosphere at a temperature of 25 °C, relative humidity of 60%, and 12 h light/12 h darkness cycle with free access to water and standard laboratory animal food pellets.

### Experimental design

**Fig. 1:**
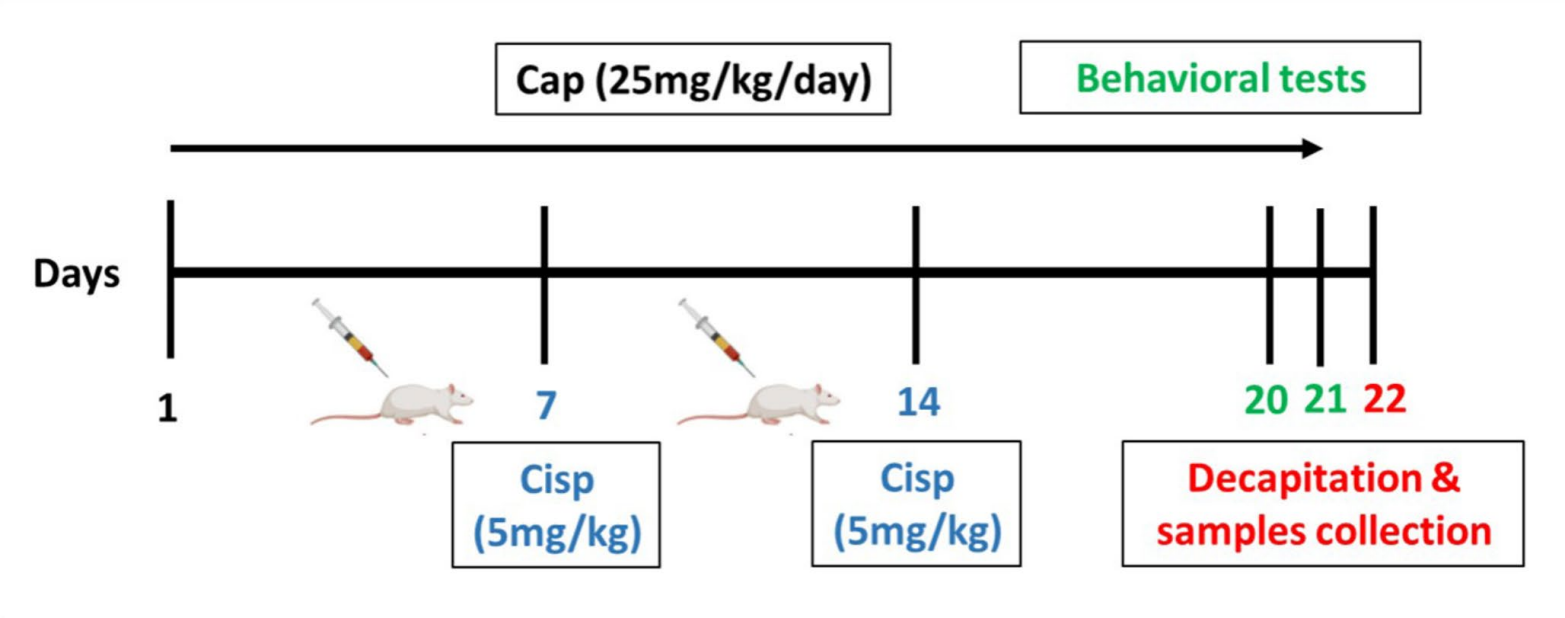
Study timeline in terms of days of administration of Cisp and Cap; Cisp, Cisplatin; Cap, Captopril

The sample size was determined based on a priori sample size calculation that was conducted using G*Power analysis. With a significance level (α) of 0.05, a power (1-β) of 90%, and an effect size of 0.5, the analysis indicated that 60 rats would be required for this study. The design of this experiment was a completely randomized design where rats were arbitrarily allocated into four groups, with 15 rats/group distributed into three cages (5 rats/cage) and were treated for 21 days in this way: rats in the first (control) and the second (cisplatin) groups were administered 0.9% saline vehicle (5 ml/kg), once daily through oral gavage. Rats in the third (captopril/cisplatin group) and fourth (captopril) groups took a single dose of captopril (25 mg/kg, by oral gavage), once daily. On days 7 and 14, the second and third groups were injected with cisplatin (5 mg/kg, i.p.), 1 h after vehicle or captopril administration, respectively. The administration of captopril, cisplatin, or vehicle was carried out in a blinded manner. The neurotoxic dose of cisplatin (Almutairi et al. 2017) and the protective dose of captopril (Boskabadi et al. 2018) were selected according to previous in-vivo experiments. Six days following the second cisplatin injection, on day 20, the training session of passive avoidance (PA) was performed. Then, on day 21, the other behavioral tests, Y-maze, test session of PA, locomotor activity, and rotarod tests were carried out. From each group, 10 rats were randomly selected from the three cages for conducting all behavioral tests. The next day following behavioral assessment, cervical dislocation was implemented for the euthanization of rats then brains were cut off and separated. The whole brains of three rats were immediately immersed in the proper buffers for histological and immunohistochemical analyses. Hippocampal and cortical tissues were dissected out from the remaining rats and kept at -80 °C for upcoming biochemical analyses. The behavioral tests, biochemical analyses, and histopathological examination were carried out in a blinded pattern. The timeline of the experiment is illustrated in Fig. 1.

### Behavioral tests

#### Y maze test

Y maze spontaneous alternation test is used to investigate the working memory following the method explained by (Miedel et al. 2017). The device is composed of a black maze with three identical arms (A, B, and C) that were intersected at an angle of 120° with 40 cm length, 15 cm height, and 8 cm width. The rats were free to move after putting them individually in the center of the apparatus for 5 min. The total arm entries (TAE) were recorded over a period of 5 min. A spontaneous alternation is identified by three consecutive accesses into three dissimilar arms, for instance (ABC, CAB, and BCA). The spontaneous alternation percentage (SAP) was then estimated by this equation: SAP = (number of alternations) / (TAE − 2) × 100.

#### Passive avoidance test

The step-through passive avoidance device (Ugo Basile, Italy) is used for investigating memory alterations according to the protocol of a previous study (Abdel-Aziz et al. 2016). The device consists of illuminated and dark sections. A grid system connected to an electric source is supplied within the bottom of the dark section. Each rat underwent a training session, followed by a test session 24 h later. In the training phase, every animal was situated in the illuminated part, and after 10 s, the sliding door between the two compartments opens and the rat enters the dark section followed by closing the door and exposing the rat to a constant electric shock of 1 mA for two seconds. During the test session, every rat was located in the light unit and the latency to enter the dark unit, without delivering an electric shock, was recorded and served as the step-through latency. At this time, the latency was recorded up to five minutes (cut-off time).

#### Locomotor activity

The locomotor activity was estimated using the locomotor activity detector (opto-Varimex-Mini Model B; Columbus Instruments, OH, USA) which operates on the infrared photocell principle. The number of interruptions of the released infrared radiation by the rat movements is counted and reflects the motor activity. The values of locomotor activity were expressed as the total number of counts per 5 min (El-Agamy et al. 2018).

#### Rotarod test

The rotarod apparatus is used to assess the rats’ balance and motor coordination on a rotating rod. Rats were initially trained on the rolling rod for 60 s at a low speed of 4 rpm. Afterward, rats were situated on the spinning drums and the time through which rats maintain balance and resist rod movement was monitored under the increased speed program (4 rpm to 40 rpm) over 2 min. The latency of rats to fall off the accelerated rotarod was determined (Liu et al. 2015).

### Histopathological examination

Following the behavior tests, brain tissues were collected and preserved in a 10% neutral paraformaldehyde solution. Tissue samples were dehydrated with gradient ethanol, cleaned in xylene, immersed in paraffin wax, and divided 5-µm thickness for hematoxylin and eosin (H & E) staining (Bancroft, J.D. and Gamble 2013). Using the light microscope and Camera: The Axio imager 2, zeiss microscopy, Germany, the H&E stained sections were examined (Abdel-latif et al. 2018).

### Brain homogenate preparation

The dissected brain tissues (hippocampi and cortices) were homogenized using ice-cooled phosphate buffer saline (PBS, 0.01 M, pH = 7.4) by the Ultra-Turrax^®^ T-25 homogenizer (IKA, Germany) to prepare 10% homogenate. Centrifugation of the homogenate was done at 4000 rpm for 15 min at 4 °C. After that, the supernatant was assembled and kept at -80 °C for further neurobiochemical analyses. Two sets of samples were used for biochemical evaluations where each set consisted of 6 samples per group in which the selection of samples occurred in a blinded manner. One set was used for spectrophotometric determination of oxidative stress markers: MDA levels and catalase activity while another set was utilized for ELISA measurements of inflammatory markers: TNF and IL-6. The samples were measured in duplicates.

### Biochemical evaluation of the oxidative status

The quantity of malondialdehyde (MDA) was measured based on the method of Mihara and Uchiyama (Uchiyama and Mihara 1978). Moreover, catalase activity was assessed in the hippocampi and cortices utilizing a detection kit purchased from Biodiagnostics Co. (Giza, Egypt) and performed in line with the company’s procedure. The colorimetric reaction was determined using the UV spectrophotometer: UV-1601 Shimadzu^®^ (Shimadzu scientific instruments, USA). Results were standardized to the brain tissue weight and presented as nmol /g tissue for MDA content and U/ g tissue for catalase activity.

### Immunohistochemical staining for glial fibrillary acid protein (GFAP) and nuclear factor kappa-B (NF-κB)

Sections from hippocampi and cortices of 5 μm thickness were dewaxed using xylene and descending ethanol concentration. Sections were washed with PBS, blocked in 10% normal serum with 5% bovine serum albumin in tris-buffered saline, and then incubated during the night at 4 °C with mouse monoclonal anti-GFAP antibody or rabbit polyclonal anti-NF-κB antibody (Thermo Fisher Scientific-Invitrogen, USA) at a 1:200 dilution. PBS was then used to wash the sections before being incubated for 20 min with biotinylated secondary antibody at a 1:500 dilution. The slides were then rinsed with PBS and counterstained with diaminobenzidine followed by hematoxylin staining. After dehydration, sections were cleaned in xylene and the immunoreactivity was pictured using a light microscope and Camera: Axio imager 2, zeiss microscopy, Germany. The acquired images were analyzed by computer-assisted image J analysis software. The immunostaining of GFAP and NF-ĸB was computed by determining the optical density (OD) in five non-overlapping fields for each rat’s hippocampal/cortical tissue section using three different rats’ brain tissues. The detailed description of the quantitative analysis method and the limitations of the immunohistochemical quantification have been supplied in **Supplementary file 1**.

### Determination of tumor necrosis factor-alpha (TNF) and interleukin-6 (IL-6) levels in hippocampal and cortical tissues

ELISA kits for measuring the contents of rat TNF (Fine Test, Cat. No. ER1393, Wuhan, China) and rat IL-6 (SunLong Biotech, Cat. No. SL0411Ra, HangZhou, China) were used in accordance with the company’s protocol. The microplate reader (ChroMate-4300, Palm City, FL) was used to read the colorimetric results of ELISAs. The results of TNF and IL-6 levels were standardized to the brain tissue weight and expressed as ng/gm tissue.

### Statistical analysis

Data parameters were displayed as mean ± SD and evaluated by one-way analysis of variance (ANOVA) followed by the Tukey–Kramer post-hoc test. The non-parametric data of step-through latency were analyzed by the Kruskal–Wallis test followed by Dunn’s post-hoc test and illustrated as median and interquartile range. The probability level less than 0.05 indicated statistical significance. All statistical analyses were carried out by InStat version 3 software and sketching graphs was performed by GraphPad Prism version 10 software (GraphPad Software Inc., USA).

## Results

### Captopril counteracted cisplatin-induced cognitive impairment and motor disturbance

**Fig. 2:**
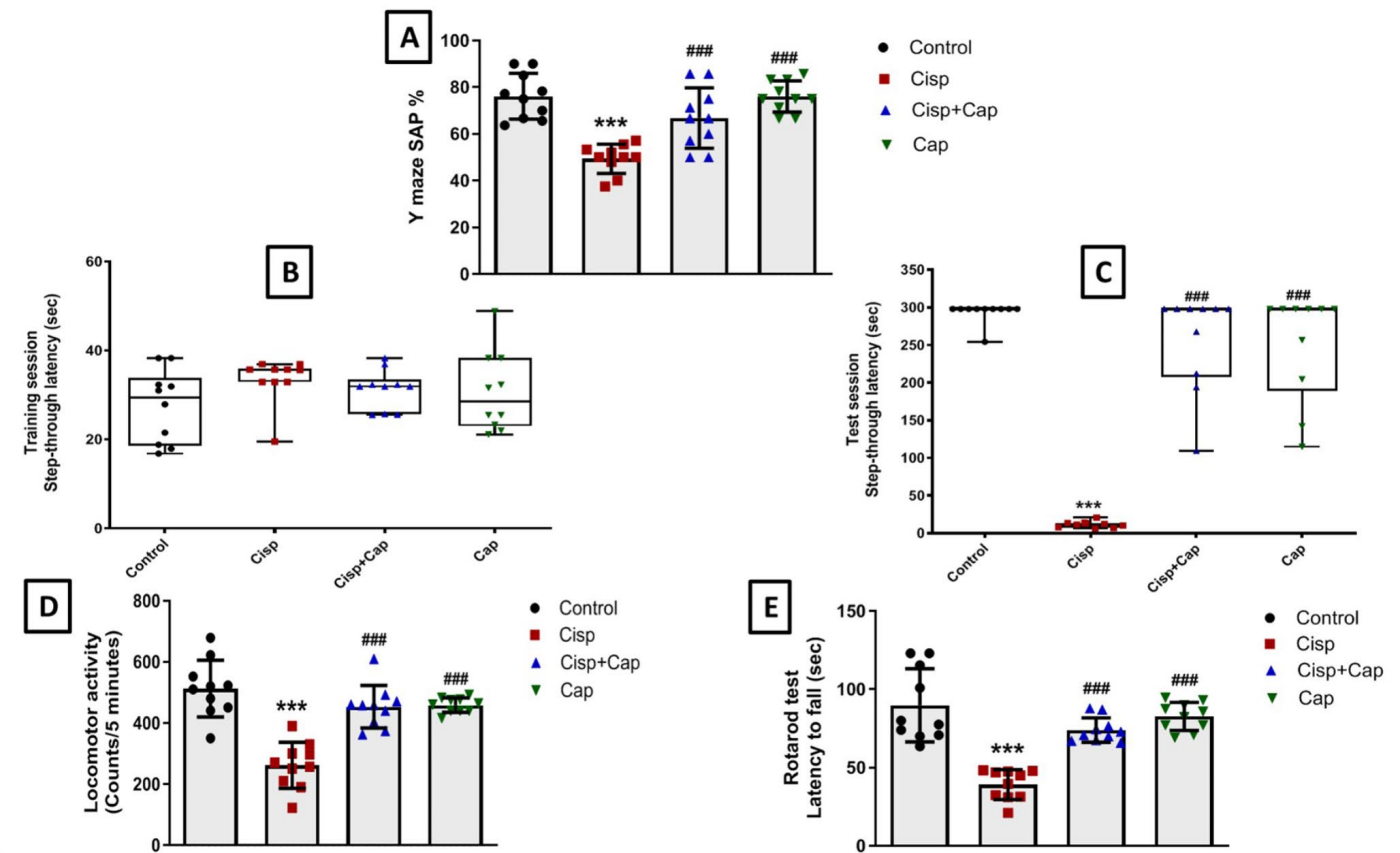
Impact of captopril treatment on cisplatin-induced behavioral changes. (**A**) Y maze spontaneous alternation percent (SAP). Data is presented as mean ± SD (*n* = 10). * or #: Statistically significant from control or cisplatin group, correspondingly such that */# at *P* < 0.05, **/## at *P* < 0.01 and ***/### at *P* < 0.001 by means of one-way ANOVA then Tukey–Kramer post hoc test. (**B**) Step-through passive avoidance training session. (**C**) Step-through passive avoidance test session. Kruskal– Wallis test was utilized for data analysis followed by Dunn’s post hoc test represented as medians and interquartile range (*n* = 10). (**D**) Locomotor activity. Data is presented as mean ± SD (*n* = 10). * or #: Statistically significant from control or cisplatin group, correspondingly such that */# at *P* < 0.05, **/## at *P* < 0.01 and ***/### at *P* < 0.001utilizing one-way ANOVA then Tukey–Kramer post hoc test. (**E**) Rotarod test latency to fall. Data is presented as mean ± SD (*n* = 10). * or #: Statistically significant from control or cisplatin group, correspondingly such that */# at *P* < 0.05, **/## at *P* < 0.01 and ***/### at *P* < 0.001 via one-way ANOVA then Tukey–Kramer post hoc test

Spatial short-term memory functions were tested through detecting SAP in Y- maze test in the four groups. Significant variations in Y-maze percent of spontaneous alternation test results were found among groups (F (3, 36) = 18.33) (Fig. 2A). Rats in the cisplatin group exhibited a significant decline in SAP by 35.2% (49.34 ± 6.25 vs. 76.14 ± 9.79 in controls, *P* < 0.0001). Conversely, captopril co-treatment enhanced short-term memory performance as shown by a significant rise in SAP by 35.4% (66.83 ± 12.94, *P* = 0.0009) compared to the cisplatin group. Additionally, there was no significant variation in SAP among the captopril-only and control groups.

Moreover, to investigate the potential long-term anti-amnestic effect of captopril on cisplatin-induced cognitive deficit, a step-through passive avoidance test was carried out for the various treated groups. According to Kruskal-Wallis test results, no statistically significant difference was observed in step-through latency among the different treatment groups during the training period (Fig. 2B). Nonetheless, the cisplatin group displayed a significant reduction in the step-through latency time during the test session (seconds) by 96% (11.75(7.45–13.5) vs. 298(298–298) in controls, *P* < 0.0001) and this obviously illustrated substantial memory dysfunction caused by cisplatin. Nevertheless, co-treatment with captopril protected against cisplatin-induced dementia as shown by returning the step-through latency near to the control level (298(207.6–298)). Captopril alone had no effect on step-through latency versus the control group (Fig. 2C**).**

Additionally, the evaluation of locomotor activity for rats (counts/ 5 min) (F (3, 36) = 24.47) showed a significant decrease in the cisplatin group by 49% (261.7 ± 75.55 vs. 512.8 ± 92.98 in controls, *P* < 0.0001). On the other hand, co-administration of captopril with cisplatin significantly elevated the locomotor activity by 73.2% (453.3 ± 69.52, *P* < 0.0001) compared to the cisplatin group. Concerning the captopril-only group, no significant variation was observed in the locomotor activity versus the control group (Fig. 2D**).**

Further, rats were assessed for their motor coordination by the rotarod test (F (3, 36) = 25.96). A significant decline in the time spent on the rotating rod (seconds) was observed in the cisplatin group by 56.5% (39.11 ± 9.58 vs. 89.83 ± 23.38 in controls, *P* < 0.0001). However, co-administration of captopril with cisplatin significantly enhanced the motor coordination of rats as evidenced by a significant rise in time spent on the rotarod by 89.3% (73.99 ± 7.86, *P* < 0.0001) versus the cisplatin group. Regarding the captopril-only group, there was no significant difference in the rotarod time in comparison with the control group (Fig. 2E**).**

### Captopril ameliorated cisplatin-induced hippocampal and cortical neurodegenerative changes

**Fig. 3:**
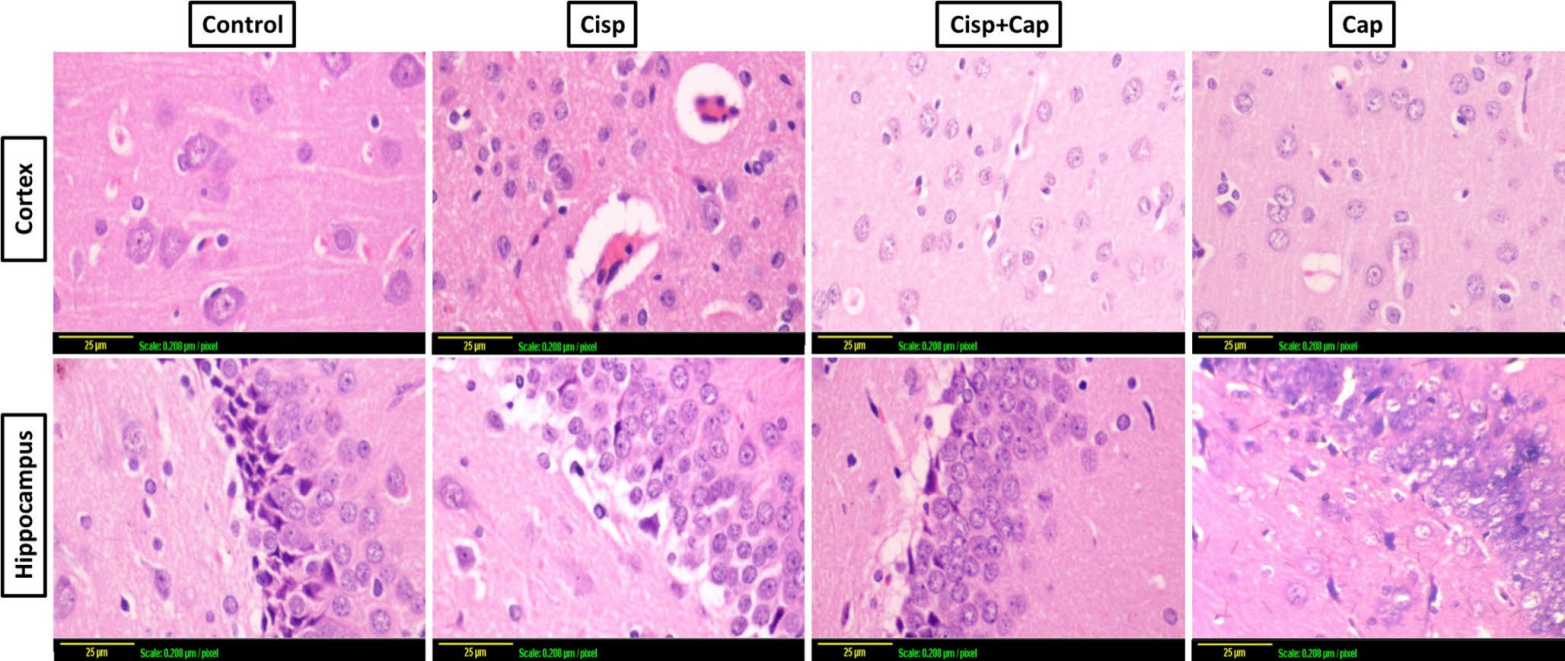
Impact of captopril treatment (25 mg/kg/day, p.o., for 21 days) on cisplatin-induced neurodegenerative alterations in the hippocampus and cortex using H and E staining (X40) (scale bar 25 μm, scale: 0.208 μm/ pixel). Cisplatin triggered nuclear pyknosis and deterioration in the neuronal cells of the hippocampus and the cortex. Alternatively, co-treatment with captopril (25 mg/kg) normalized the hippocampal and cortical histological architecture

Light microscopy analysis of the cerebral cortex and hippocampus of the control group and captopril only-treated group, revealed normal histological architecture without any significant pathological alterations. The cerebral cortex was made up of several layers of neuronal cells containing oval or rounded nuclei with notable nucleoli encompassed by scanty basophilic cytoplasm. Moreover, the hippocampus indicated normal histological structure appeared as layers of compact granular cells with dark nuclei. Additionally, glial cells and pyramidal cells were identified in the molecular layer. Alternatively, the cerebral cortex of the cisplatin group indicated neuronal degeneration which appeared as darkly stained neurons encompassed by per-cellular halo spaces. Apoptosis of some neuronal cells with condensed and clumped nuclear chromatin associated with focal gliosis was noticed. Moreover, obvious perivascular edema was detected. In addition, the hippocampal section showed cellular disorganization and shrinkage of large pyramidal cells with great vacuolations in granular cell layers. Noteworthy, the cerebral cortex of the cisplatin-treated rats co-treated with captopril, showed normal histological structure without any marked pathological changes. The neuronal cells have round nuclei with notable nucleoli encompassed by scanty basophilic cytoplasm. Furthermore, the hippocampus indicated normal arrangement of granular cells with dark nuclei (Fig. 3).

### Captopril prevented cisplatin-induced oxidative stress in both hippocampal and cortical tissues

**Fig. 4:**
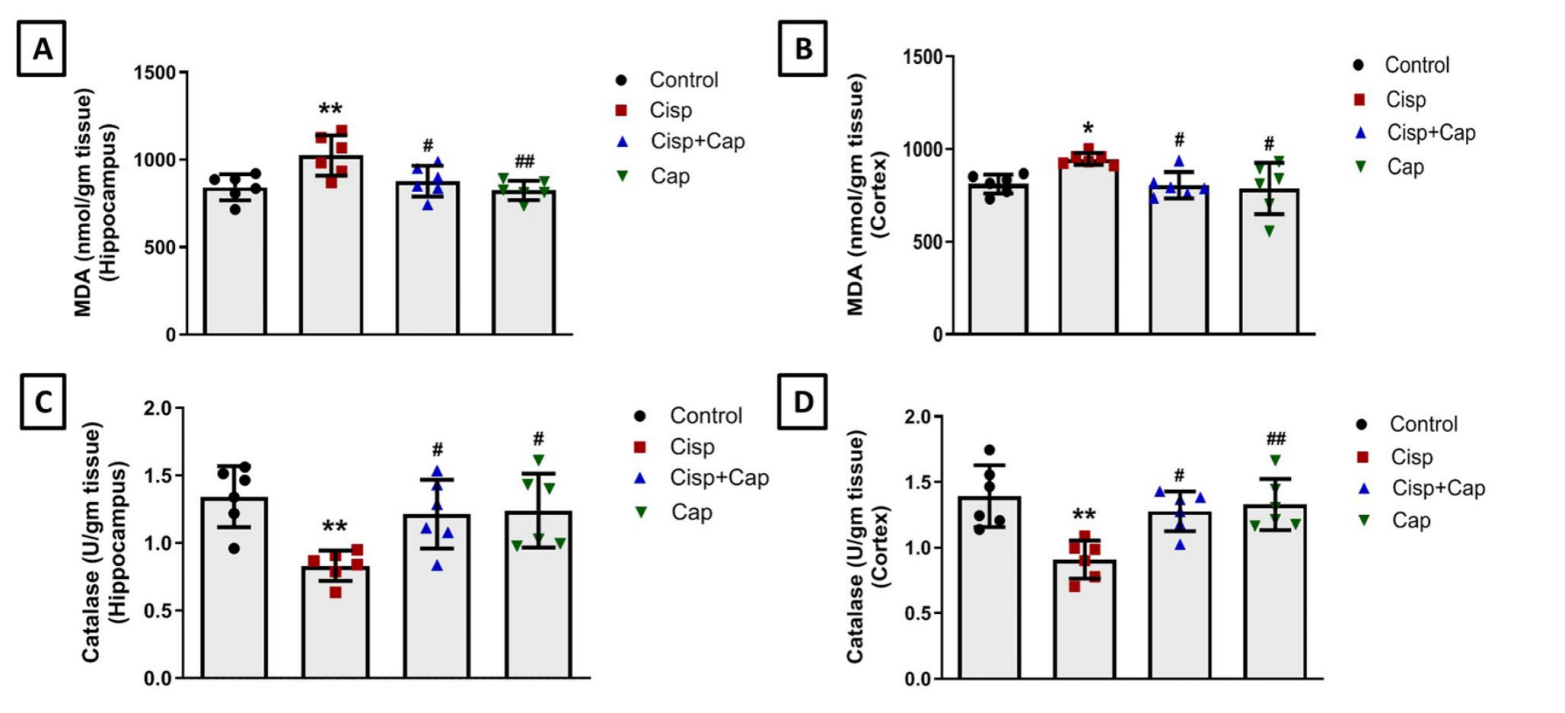
Impact of captopril on cisplatin-induced oxidative stress in the hippocampus and cortex. MDA levels in hippocampus (**A**) and cortex (**B**). Catalase activity in hippocampus (**C**) and cortex (**D**). Data is illustrated as mean ± SD (*n* = 6). * or #: Statistically significant from control or cisplatin group, correspondingly such that */# at *P* < 0.05, **/## at *P* < 0.01 and ***/### at *P* < 0.001 applying one-way ANOVA then Tukey–Kramer post hoc test

Cisplatin-mediated oxidative stress in the hippocampal and cortical tissues was evaluated by assessing MDA levels and catalase activity. As shown in Fig. 4, no significant alterations in oxidative stress markers were detected in brain tissues of captopril alone-treated rats in comparison with the control group. Nevertheless, evaluation of the lipid peroxidation state of cisplatin-treated rats showed significant elevation of MDA levels (nmol/gm tissue) by 21.7% (1025 ± 115.4 vs. 842 ± 74.49 in controls, *P* = 0.0076) and 16.7% (947.1 ± 31.81 vs. 811.7 ± 51.54 in controls, *P* = 0.0496) in the hippocampus (F (3, 20) = 6.68) and cortical (F (3, 20) = 4.6) tissues, respectively. On the contrary, captopril co-treatment significantly decreased hippocampal and cortical MDA levels by 14.4% (877.5 ± 88.13, *P* = 0.0361) and 15% (805.5 ± 70.87, *P* = 0.0382) respectively, versus cisplatin group (Fig. 4A and B).

Moreover, administration of cisplatin significantly decreased catalase activity (U/gm tissue) in the hippocampal (F (3, 20) = 5.92) and cortical (F (3, 20) = 8.24) tissues by 38% (0.83 ± 0.11 vs. 1.34 ± 0.23 in controls, *P* = 0.0043) and 34.5% (0.91 ± 0.14 vs. 1.39 ± 0.24 in controls, *P* = 0.0011), respectively. In contrast, co-treatment with captopril caused a significant increase in hippocampal catalase activity by 45.8% (1.21 ± 0.26, *P* = 0.0378) and showed a significant elevation in the cortical catalase activity by 40.7% (1.28 ± 0.15, *P* = 0.0127) in comparison with the cisplatin group (Fig. 4C and D).

### Captopril ameliorated cisplatin-induced neuroinflammation

Immunohistochemical staining was utilized for evaluation of the expression of GFAP and NF-κB, while TNF and IL-6 concentrations were assessed by ELISA technique.

**Fig. 5:**
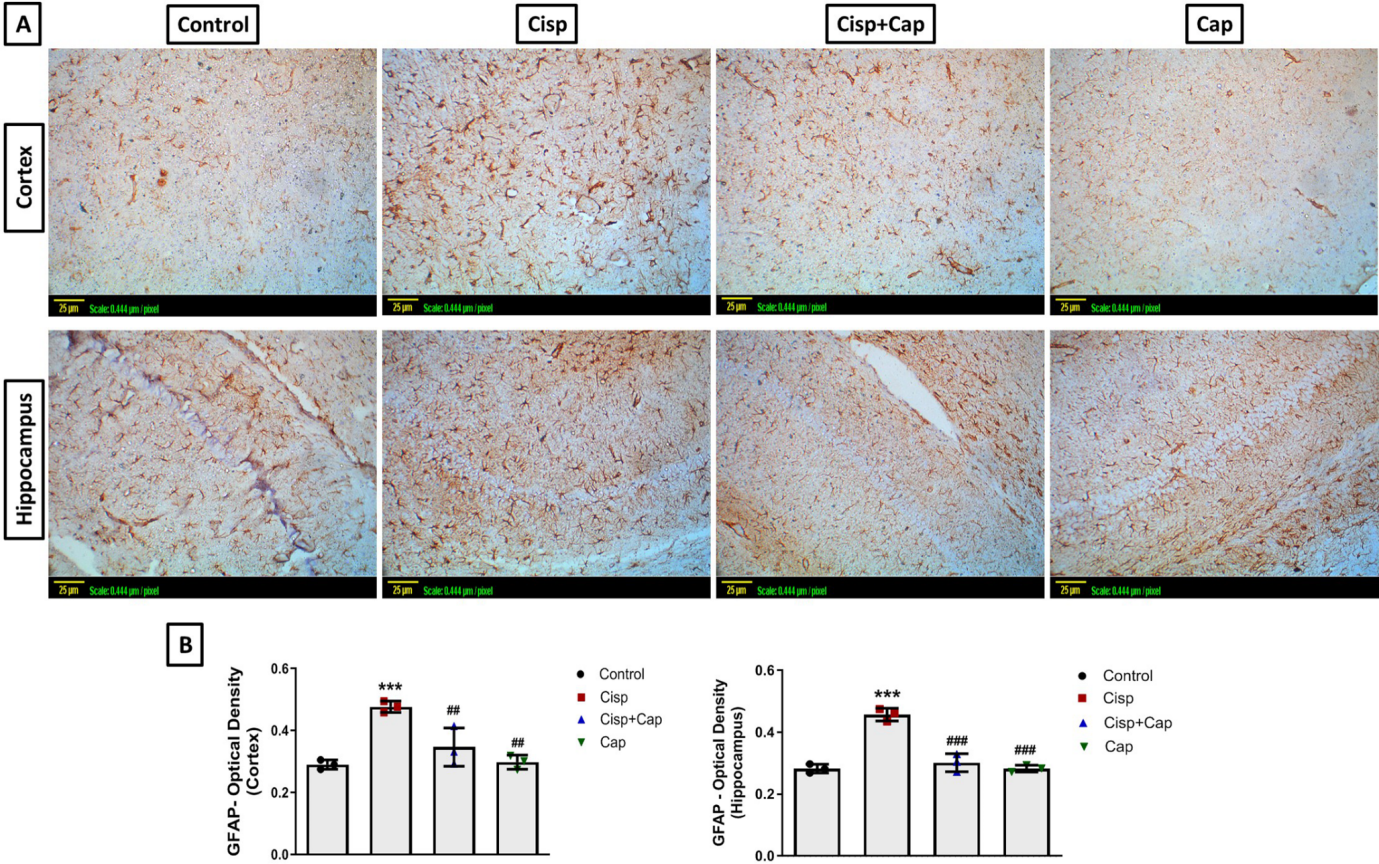
Impact of captopril on GFAP-immunostaining in the hippocampus and cortex. (**A**) Representative photomicrographs of GFAP-immunostained sections of hippocampus and cortex (X20) (scale bar 25 μm, scale: 0.444 μm/pixel), indicating marked brown staining in the cisplatin group. (B) Bar chart representation for the optical density in the different groups. Data is indicated as mean ± SD (*n* = 3). * or #: Statistically significant from control or cisplatin group, correspondingly such that */# at *P* < 0.05, **/## at *P* < 0.01 and ***/### at *P* < 0.001 via one-way ANOVA then Tukey–Kramer post hoc test

**Fig. 6:**
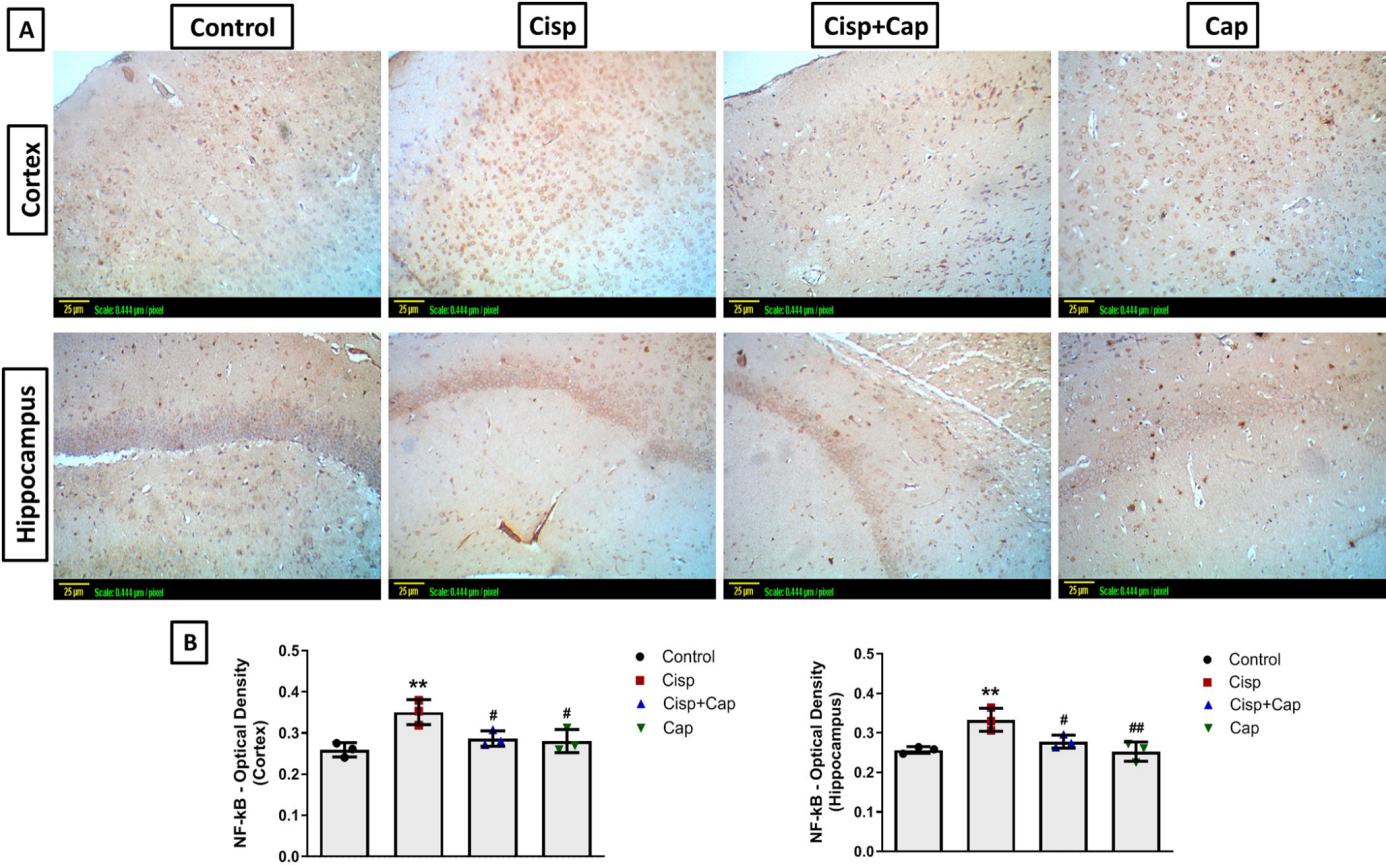
Impact of captopril on NF-κB-immunostaining in the hippocampus and cortex. (**A**) Representative photomicrographs of NF-κB -immunostained sections of the hippocampus and cortex (X20) (scale bar 25 μm, scale: 0.444 μm/pixel), indicating remarkable brown staining in the cisplatin group. (**B**) Bar chart representation for the optical density of NF-κB staining in the various groups. Data is indicated as mean ± SD (*n* = 3). * or #: Statistically significant from control or cisplatin group, correspondingly such that */# at *P* < 0.05, **/## at *P* < 0.01 and ***/### at *P* < 0.001 by means of one-way ANOVA then Tukey–Kramer post hoc test

Minimum immunostaining was shown in the hippocampal and cortical tissue sections of the control group and captopril-only group, concerning both GFAP and NF-κB. However, the cisplatin group revealed raised expression of GFAP and NF-κB as presented by the marked brown staining versus the control group for both markers. The (OD) of the stained areas was used to quantify the immunohistochemical staining utilizing the Image J analysis software (Figs. 5 and 6). In case of cortical GFAP expression (F (3, 8) = 18.15), cisplatin caused a significant elevation in OD by 65.5% (0.48 ± 0.018 vs. 0.29 ± 0.015 in controls, *P* = 0.0008). Moreover, for hippocampal GFAP expression (F (3, 8) = 54.48), cisplatin caused a significant elevation in OD by 64.3% (0.46 ± 0.02 vs. 0.28 ± 0.014 in controls, *P* < 0.0001). In case of cortical NF-κB expression (F (3, 8) = 7.88), cisplatin caused a significant increase in OD by 34.6% (0.35 ± 0.03 vs. 0.26 ± 0.017 in controls, *P* = 0.0075). Additionally, for hippocampal NF-κB expression (F (3, 8) = 9.23), cisplatin caused a significant increase in OD by 26.9% (0.33 ± 0.029 vs. 0.26 ± 0.009 in controls, *P* = 0.0094).

Alternatively, captopril co-treatment significantly hampered this elevation in OD for both markers such that, for cortical GFAP expression, captopril co-treatment caused a significant decline by 27.1% (0.35 ± 0.062, *P* = 0.0081) versus the cisplatin group. Moreover, for hippocampal GFAP expression, captopril co-treatment caused a significant decline by 34.8% (0.3 ± 0.03, *P* < 0.0001) versus the cisplatin group. In case of cortical NF-κB expression, captopril co-treatment caused a significant decrease by 17.1% (0.29 ± 0.019, *P* = 0.0481) versus the cisplatin group. Furthermore, for hippocampal NF-κB expression, captopril co-treatment caused a significant decrease by 15.2% (0.28 ± 0.017, *P* = 0.0498) versus the cisplatin group.

**Fig. 7:**
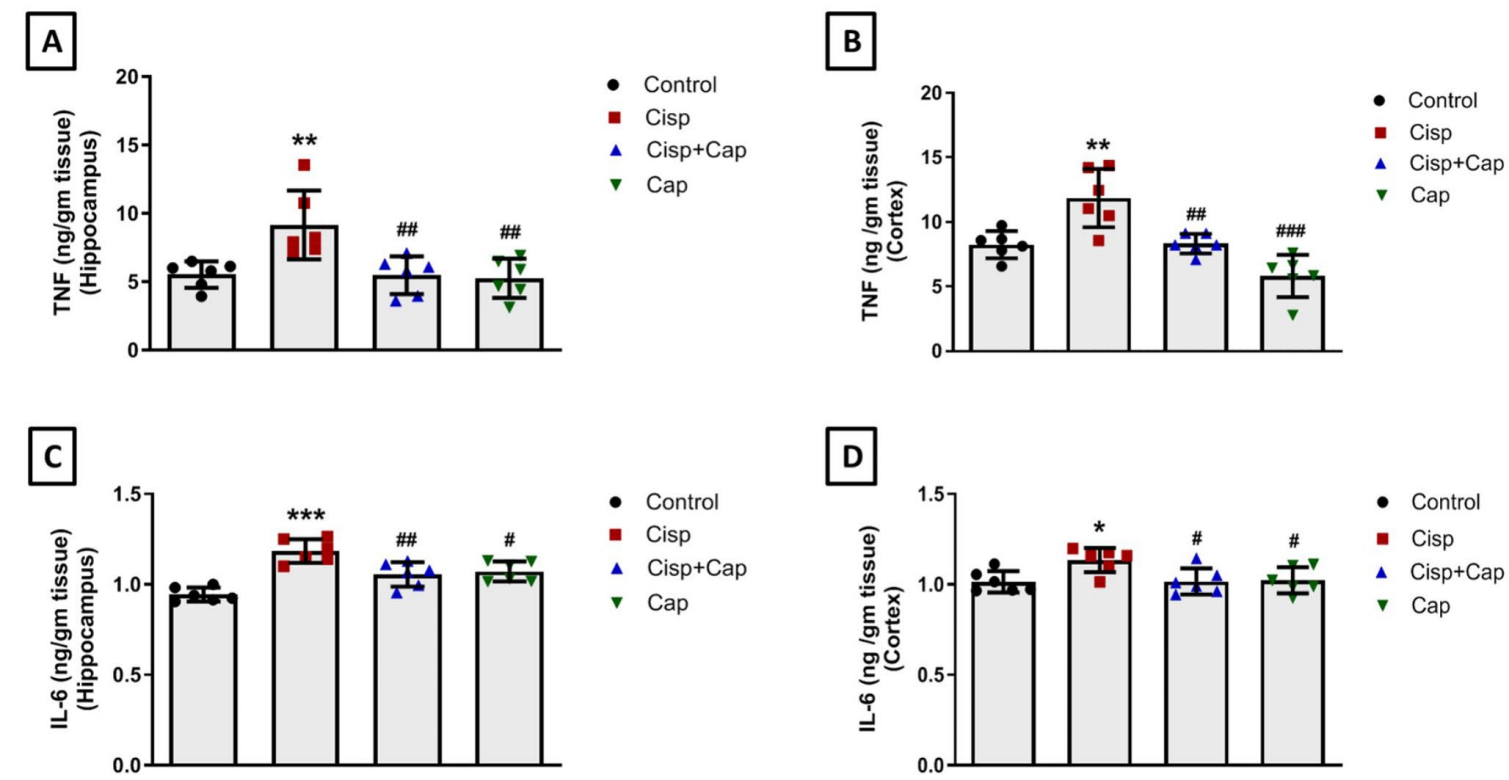
Impact of captopril on cisplatin-mediated alterations in TNF and IL-6 levels. TNF levels in the hippocampus (**A**) and cortex (**B**). IL-6 levels in the hippocampus (**C**) and cortex (**D**). Data is illustrated as mean ± SD (*n* = 6). * or #: Statistically significant from control or cisplatin group, correspondingly such that */# at *P* < 0.05, **/## at *P* < 0.01 and ***/### at *P* < 0.001 by means of one-way ANOVA then Tukey–Kramer post hoc test

Cisplatin-induced neuroinflammation was further confirmed by measurement of the cytokines levels; TNF (F (3, 20) = 7.5) and IL-6 (F (3, 20) = 17.43). Cisplatin showed a significant elevation in the levels of TNF (ng/gm tissue) by 65.6% (9.16 ± 2.52 vs. 5.53 ± 0.96 in controls, *P* = 0.0063) in the hippocampus and 43.8% (11.85 ± 2.26 vs. 8.24 ± 1.05 in controls, *P* = 0.0032) in the cortex. On the contrary, a significant reduction of hippocampal and cortical TNF levels by 40.3% (5.47 ± 1.38, *P* = 0.0056) and 29.8% (8.32 ± 0.76, *P* = 0.0039) respectively, was obtained upon co-treatment with captopril versus the cisplatin group (Fig. 7A and B).

In addition, significant elevations in the levels of IL-6 (ng/gm tissue) were obtained in the cisplatin group by 26.6% (1.19 ± 0.065 vs. 0.94 ± 0.039 in controls, *P* < 0.0001) and 11.8% (1.14 ± 0.067 vs. 1.02 ± 0.059 in controls, *P* = 0.0302) in the hippocampal and cortical tissues respectively. Conversely, co-treatment with captopril caused a significant reduction in hippocampal and cortical IL-6 levels by 10.9% (1.06 ± 0.067, *P* = 0.0047) and 10.4% (1.02 ± 0.073, *P* = 0.0326) correspondingly, versus the cisplatin group (Fig. 7C and D**).**

**Fig. 8:**
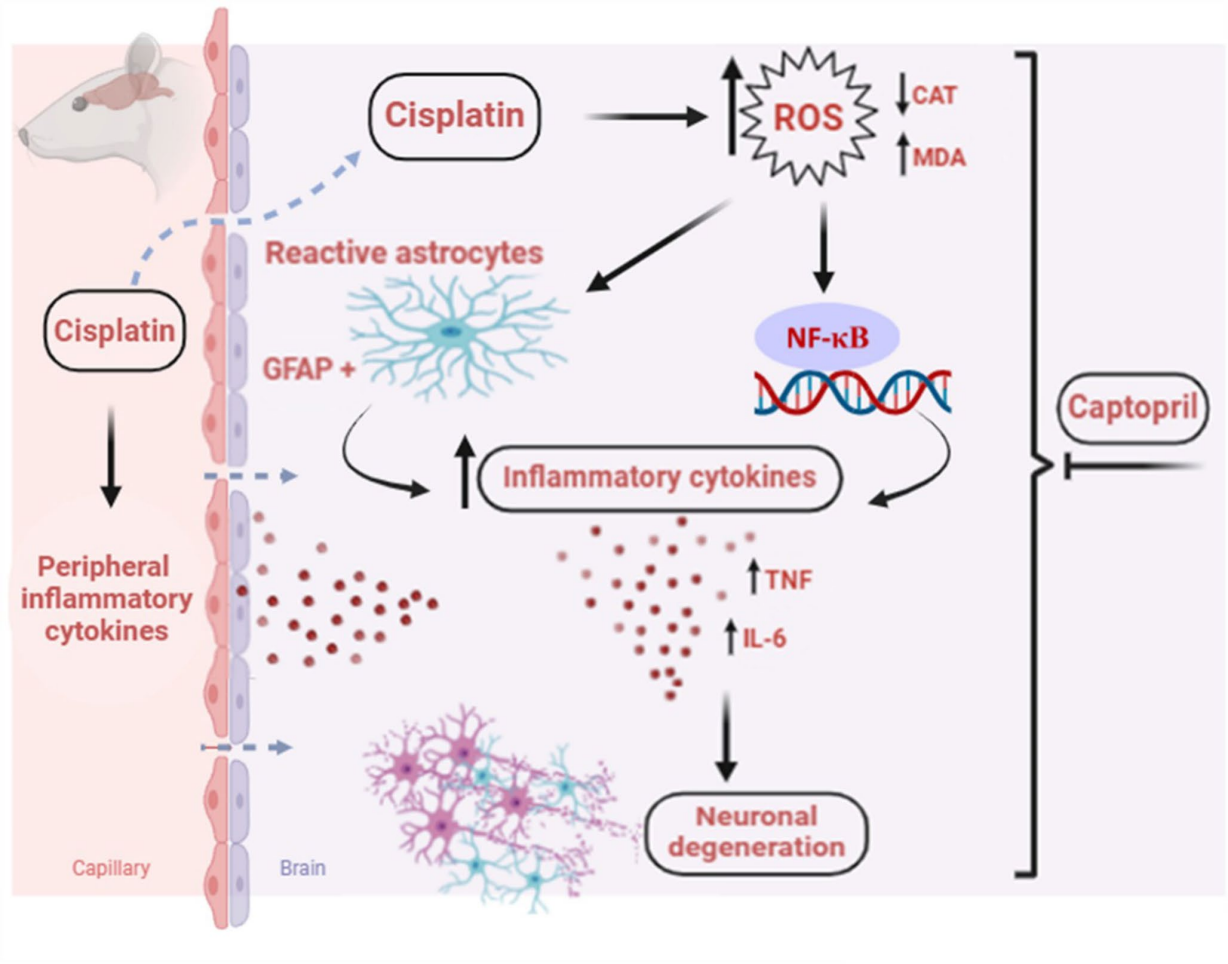
A schematic diagram demonstrating the potential mechanisms for captopril’s protective effect versus cisplatin-induced chemobrain in rats. This figure was designed by means of Biorender (publication agreement number: DQ277ZOIT1)

## Discussion

Cisplatin is a broadly used chemotherapeutic agent in the clinical setting for treating a diversity of pediatric and adult malignancies (Romani 2022). Nonetheless, its clinical usefulness is restricted due to various deleterious harmful effects and toxicities to normal tissues (Qi et al. 2019). Both experimental and clinical reports have offered distinct evidence for cisplatin-induced learning and memory impairment (Amidi et al. 2017; Mahmoud et al. 2023). Recently, captopril has attracted great attention in the discipline of neurology because of its appealing neuroprotective capabilities that have been verified in various experimental studies (Sonsalla et al. 2013; Tao et al. 2018). Our study stated that captopril significantly improved cognitive performance in rats intoxicated with cisplatin. Next, we employed different biomarkers to investigate the mechanism of neuroprotection and we reported that the antioxidant and anti-inflammatory activities of captopril are the key players in attenuating hippocampal and cortical neuronal damage and cognitive impairment associated with cisplatin. Intriguingly, our results paved the way for the repositioning of captopril in managing chemobrain, especially for hypertensive cancer patients. Likewise, such repurposing scenario for antihypertensive drugs has been implicated in the study of (Ali et al. 2022) using the antihypertensive amiloride against doxorubicin-induced chemobrain in rats.

It has been reported that the penetration of platinum analogs, including cisplatin, into the blood-brain barrier (BBB) and cerebrospinal fluid (CSF) is restricted (Jacobs et al. 2005, 2010). Nevertheless, the penetration of cisplatin into BBB is favored by multiple confounding factors that impair the BBB integrity including brain tumors, and the massive discharge of peripheral proinflammatory cytokines driven by cisplatin (dos Santos et al. 2020). Indeed, several preclinical studies have illustrated that chemotherapeutic agents trigger a rise in the peripheral inflammatory cytokines that can penetrate BBB provoking further discharge of local pro-inflammatory cytokines and ROS in the brain and eventually exaggerating the central oxidative and neuroinflammatory signals (Lomeli et al. 2021; Jaiswara and Shukla 2023). Besides, these pro-inflammatory cytokines disrupt the BBB’s integrity permitting the crossing of chemotherapeutic agents through BBB hence prompting a cascade of local inflammatory responses in the brain thus aggravating central neurotoxicity and neurodegeneration (Wardill et al. 2016). Moreover, ample evidence has disclosed that cisplatin mediates pathological changes and significant disruption in the hippocampus which is critical for context fear conditioning resulting in impaired context memory (Zhou et al. 2016; Lomeli et al. 2017; Mahmoud et al. 2023). Hence, the cognitive function of cisplatin-treated rats was evaluated through conducting Y-maze and passive avoidance tests which are common behavior tests for memory and learning. Cisplatin intoxication caused a significant decline in spatial working memory as evident by the marked drop in Y-maze spontaneous alternation. In addition, cisplatin negatively impacted memory retention as shown by significantly declining the step-through latency in the passive avoidance test. These results confirmed the neurotoxic effect of cisplatin reflected by diminished learning and memory capacity. Besides, it is noteworthy that cerebellar cortex, which is markedly involved in manipulating motor balance and coordination, is extensively damaged by cisplatin (Avella et al. 2006). In this context, cisplatin-treated rats in this study showed a significant decline in locomotor activity as well as muscle weakness as evident by significantly reducing the latency to fall off the rotarod drum. In accordance with neurobehavioral findings, histopathological examination showed obvious pathological changes in the hippocampus and cortex of the cisplatin group, as neuronal degeneration and apoptosis of neuronal cells, that is compatible with former experiments (Arafa and Atteia 2020; Saral et al. 2021). Here, we stated that captopril co-treatment hampered cisplatin-induced neuronal damage on the levels of both neurobehavioral tests and histopathological changes implicating its notable neuroprotective effects. Likewise, growing evidence stated that the use of ACEI which are able to penetrate BBB is accompanied by enhanced cognitive performance in elderly patients with AD and dementia (Gao et al. 2013; Rygiel 2016).

Next, we investigated the underlying mechanisms for the neuroprotective effect of captopril *via* assessment of the oxidative stress and neuroinflammatory pathways implicated in neurotoxicity. It’s well documented that oxidative stress performs a crucial function in the pathogenesis of neuronal damage and memory impairment associated with cisplatin (Abdel-Wahab and Moussa 2019; Mahmoud et al. 2023). The elevated oxidative stress has been further correlated with mood, learning, and, cognitive dysfunction (Jangra et al. 2016; Kumburovic et al. 2019). Given that cisplatin is an alkylating anticancer drug, it elicits direct damage of DNA especially the mitochondrial one interfering with mitochondrial oxidative metabolism eventually leading to immense production of ROS which then overwhelms the antioxidant defense system (Kleih et al. 2019). This is thought of great concern as brain is especially sensitive to pathological damage from ROS due to its high oxygen utilization, the abundant lipid contents beside its scanty antioxidant defenses compared to other organs (Cenini et al. 2020). Our results indicated marked elevation in oxidative status in the hippocampus and cortex of the cisplatin group as shown by the raised level of lipid peroxidation product, MDA, and consequently diminished activity of antioxidant catalase enzyme. These findings agreed with a previous report indicating that the neurodegeneration caused by cisplatin could be due to rising lipid peroxidation and impaired antioxidant enzymes’ activities leading to a decline in the antioxidant defense (Almutairi et al. 2017). Interestingly, it was obvious that captopril co-administration abrogated MDA production and restored the catalase antioxidant effect in both cortical and hippocampal tissues. In agreement with our findings, the antioxidant activity of captopril was documented to be involved in neuroprotection in different in-vivo models of cognitive dysfunction (Abareshi et al. 2016; Akbari et al. 2019). The ROS scavenging effect of captopril is suggested to be related to the terminal sulfhydryl group in its structure (Kalia et al. 2007). Therefore, the current study concluded that the antioxidant potential of captopril could contribute to its protective effects against cisplatin neurotoxicity.

Besides the excessive production of free radicals, cisplatin mediates behavioral malformations *via* activation of neuroinflammatory cascades (Chen et al. 2019). Notably, oxidative stress is one of the most critical triggering factors for neuroinflammatory processes (Solleiro-Villavicencio and Rivas-Arancibia 2018). Cisplatin-induced ROS activates the transcription factor NF-κB which is responsible for the transcription machinery of genes coding for several proinflammatory mediators (Lingappan 2018). In the inactive state, NF-κB is isolated in the cytoplasm by attaching to its inhibitory protein, IκB (Liu et al. 2017). Following stimulation by ROS, IκB is phosphorylated and then degraded permitting NF-κB to translocate into the nucleus commencing the transcription of an array of pro-inflammatory genes (Yu et al. 2020). Indeed, these inflammatory mediators could further stimulate more ROS production in a vicious cycle of oxidative stress-inflammation signaling (Elmarakby and Sullivan 2012**).** The proinflammatory cytokines TNF, and IL-6 are prominent inducers of cisplatin neurotoxicity (Akman et al. 2015; Hassan et al. 2024). Clinically, chemotherapy protocols including cisplatin caused a marked increase in plasma levels of both cytokines which disrupt the BBB integrity creating a crosstalk circuit among central neuroinflammation and peripheral inflammation (Fernandez et al. 2020). Furthermore, the disruption in BBB integrity by the inflammatory cytokines allows more penetration of chemotherapeutic agents into the brain thus aggravating central neurotoxicity and neurodegeneration (Wardill et al. 2016). Moreover, the up-regulation of TNF can further stimulate the transcriptional machinery of NF-κB further exacerbating neuroinflammation and hence neuronal damage (Lawrence 2009). In the current study, cisplatin administration caused upregulation of NF-κB expression alongside with marked elevation in TNF and IL-6 amounts in the hippocampus and cortex indicating marked neuroinflammation. In accordance with our findings, several lines of evidence confirmed the pathological role of NF-κB-dependent inflammatory reactions in chemobrain (Bagnall-Moreau et al. 2019). Of interest, the results of this research showed the beneficial anti-inflammatory properties of captopril since it caused a decline in TNF and IL-6 levels besides reduced NF-κB expression in the rats’ hippocampal and cortical tissues. Similarly, captopril decreased the deleterious impact of TNF on the BBB integrity in addition to repressing IL-6 levels following lipopolysaccharide intoxication in an experimental model of learning and cognitive deficits in rats (Abareshi et al. 2016, 2019). Moreover, it was reported that captopril enhanced analgesic effects of morphine and prevented its tolerance through inhibiting NF-κB -mediated neuroinflammation (Taskiran and Avci 2021). Thus, the existing experiment supported the anti-inflammatory potential of captopril in attenuating neuroinflammation caused by cisplatin.

Among the glial cell population, oligodendrocytes are considered the most frequent type followed by astrocytes then the microglia represent the least proportion of glial cells in different brain areas (von Bartheld et al. 2016; Hannon et al. 2024). Astrocytes play crucial roles in preserving neuronal hemostasis under healthy conditions (Sofroniew 2020**).** They are essential regulators of the brain function *via* controlling synaptic transmission, cerebral microcirculation, neurovascular coupling, besides maintaining the BBB integrity (Kimelberg and Nedergaard 2010; Barreto et al. 2011; Santos and Pyter 2018). However, under pathological conditions, astrocytes acquire reactive phenotype in response to various neurotoxic insults then substantially boost neuroinflammation through massive release of ROS and pro-inflammatory molecules (Carson et al. 2006). Thereafter, a state of astrogliosis is created accompanied by excessive downstream production of diverse neurotoxic mediators leading to a vicious cycle of detrimental oxidative injury and neuroinflammation which further exacerbates neuronal loss (Baune et al. 2012; Cerbai et al. 2012). Indeed, GFAP is among the principal indicators for the activation of astrocytes due to brain injury and stress. Hence, one of the cornerstone markers for astrogliosis is the upregulated expression of GFAP (De Oliveira et al. 2016; Zhang et al. 2017). In our study, immunohistochemical staining was performed to evaluate GFAP expression and the results showed significant elevation in GFAP protein expression in the hippocampus and cortex of the cisplatin group that in turn ensures the progression of the neuroinflammatory process. Consistent with our findings, several studies have highlighted the serious roles of reactive astrocytes in promoting neuroinflammation and cognitive dysfunction in AD (Ferrari-Souza et al. 2022; Rodríguez-Giraldo et al. 2022). Moreover, GFAP is identified as a crucial biomarker for the diagnosis and prognosis of AD (Kim et al. 2023). Interestingly, co-treatment with captopril stabilized the GFAP levels in both hippocampus and cortex of rats intoxicated with cisplatin. These findings were in line with prior findings illustrating that captopril opposes astrocyte and microglial activation stimulated by various deleterious factors. It was found that captopril abrogated epilepsy-induced upregulation and abnormal contact between astrocytes and microglia in a rat model of status epilepticus and cognitive impairment (Dong et al. 2022). Furthermore, in an experimental model of PD, captopril reduced the activation of microglia in the substantia nigra of rats (Sonsalla et al. 2013). These findings further confirm that captopril can repress neuroinflammation associated with cisplatin-induced cognitive decline. Notably, these worthy neuroprotective effects of captopril could be also linked with angiotensin-converting enzyme (ACE) inhibition as it was reported that elevated levels of angiotensin II (ANG II) are associated with neuronal oxidative injury and inflammatory responses beside deficient cerebral blood flow culminating in detrimental effects on cognitive function (Mogi et al. 2012). Moreover, upraised activity of neuronal ACE was reported in the brains of AD patients (Miners et al. 2009). Accordingly, this supports the notion that diminishing neuronal ANG II levels through ACE inhibition could be an imperative target for neuroprotection against chemobrain. To sum up, the current study gives preclinical proof that captopril could represent a promising repurposed neuroprotective and anti-amnestic agent against cisplatin-induced cognitive deficit. Such neuroprotective potential is attributable to guarding against neuronal oxidative stress and suppressing neuroinflammation through suppressing NF-κB dependent pro-inflammatory cytokine production besides, hindering astrocyte activation thereby downgrading GFAP expression. These effects are illustrated in Fig. 8.

## References

[CR2] Abareshi A, Hosseini M, Beheshti F et al (2016) The effects of captopril on lipopolysaccharide induced learning and memory impairments and the brain cytokine levels and oxidative damage in rats. Life Sci 167:46–56. 10.1016/J.LFS.2016.10.026 27794490 10.1016/j.lfs.2016.10.026

[CR1] Abareshi A, Anaeigoudari A, Norouzi F et al (2019) The effects of captopril on lipopolysaccharide-induced sickness behaviors in rats. Veterinary Res Forum 10:199–205. 10.30466/VRF.2018.90760.2198 10.30466/vrf.2018.90760.2198 PMC6828174 31737228

[CR3] Abdel-Aziz AK, Mantawy EM, Said RS, Helwa R (2016) The tyrosine kinase inhibitor, sunitinib malate, induces cognitive impairment in vivo via dysregulating VEGFR signaling, apoptotic and autophagic machineries. Exp Neurol 283:129–141. 10.1016/J.EXPNEUROL.2016.06.004 27288242 10.1016/j.expneurol.2016.06.004

[CR4] Abdel-latif RG, Heeba GH, Taye A, Khalifa MMA (2018) Lixisenatide, a novel GLP-1 analog, protects against cerebral ischemia/reperfusion injury in diabetic rats. Naunyn-Schmiedeberg’s Archives Pharmacol 391:705–717. 10.1007/S00210-018-1497-1 10.1007/s00210-018-1497-1 29671019

[CR5] Abdel-Wahab WM, Moussa FI (2019) Neuroprotective effect of N-acetylcysteine against cisplatin-induced toxicity in rat brain by modulation of oxidative stress and inflammation. Drug Des Devel Ther 13:1155–1162. 10.2147/DDDT.S191240 31043768 10.2147/DDDT.S191240 PMC6469471

[CR6] Akbari HR, Beheshti F, Sadeghnia HR et al (2019) The effects of captopril on learning and memory impairment induced by scopolamine in rats: anti-oxidative effects. Physiol Pharmacol 23:91–100

[CR7] Akman T, Akman L, Erbas O et al (2015) The preventive effect of oxytocin to cisplatin-induced neurotoxicity: an experimental rat model. Biomed Res Int 2015:2015:167235. 10.1155/2015/167235 25688351 10.1155/2015/167235 PMC4320931

[CR8] Ali AE, Elsherbiny DM, Azab SS, El-Demerdash E (2022) The diuretic amiloride attenuates doxorubicin-induced chemobrain in rats: behavioral and mechanistic study. Neurotoxicology 88:1–13. 10.1016/J.NEURO.2021.10.002 34656704 10.1016/j.neuro.2021.10.002

[CR9] Almutairi MM, Alanazi WA, Alshammari MA et al (2017) Neuro-protective effect of rutin against cisplatin-induced neurotoxic rat model. BMC Complement Altern Med 17:1–9. 10.1186/S12906-017-1976-9 28962559 10.1186/s12906-017-1976-9 PMC5622464

[CR10] Amidi A, Hosseini SMH, Leemans A et al (2017) Changes in Brain Structural Networks and Cognitive functions in Testicular Cancer patients receiving cisplatin-based chemotherapy. J Natl Cancer Inst 109(12). 10.1093/JNCI/DJX085 10.1093/jnci/djx085 29617869

[CR11] Arafa MH, Atteia HH (2020) Protective role of Epigallocatechin Gallate in a rat model of Cisplatin-Induced cerebral inflammation and oxidative damage: impact of modulating NF-κB and Nrf2. Neurotox Res 37:380–396. 10.1007/S12640-019-00095-X 31410684 10.1007/s12640-019-00095-x

[CR12] Avella D, Pisu MB, Roda E et al (2006) Reorganization of the rat cerebellar cortex during postnatal development following cisplatin treatment. Exp Neurol 201:131–143. 10.1016/J.EXPNEUROL.2006.03.034 16806181 10.1016/j.expneurol.2006.03.034

[CR13] Bagnall-Moreau C, Chaudhry S, Salas-Ramirez K et al (2019) Chemotherapy induced cognitive impairment is associated with increased inflammation and oxidative damage in the hippocampus. Mol Neurobiol 56:7159–7172. 10.1007/S12035-019-1589-Z 30989632 10.1007/s12035-019-1589-z PMC6728167

[CR14] Bancroft JD, Gamble M (2013) Theory and Practice of Histological Techniques. 7th Edition, Churchill Livingstone of Elsevier, Philadelphia, 172–186

[CR15] Barreto G, White RE, Ouyang Y et al (2011) Astrocytes: targets for Neuroprotection in Stroke. Cent Nerv Syst Agents Med Chem 11:164–173. 10.2174/187152411796011303 21521168 10.2174/187152411796011303 PMC3167939

[CR16] Baune BT, Camara M, Lou, Eyre H et al (2012) Tumour necrosis factor - ALPHA mediated mechanisms of cognitive dysfunction. Translational Neurosci 3:263–277. 10.2478/S13380-012-0027-8/XML

[CR17] Boskabadi J, Mokhtari-Zaer A, Abareshi A et al (2018) The effect of captopril on lipopolysaccharide-induced lung inflammation. Exp Lung Res 44:191–200. 10.1080/01902148.2018.1473530 29847180 10.1080/01902148.2018.1473530

[CR18] Brown A, Kumar S, Tchounwou PB (2019) Cisplatin-based chemotherapy of human cancers. J cancer Sci Therapy 11(4):97 PMC7059781 32148661

[CR19] Carson MJ, Cameron Thrash J, Walter B (2006) The cellular response in neuroinflammation: the role of leukocytes, microglia and astrocytes in neuronal death and survival. Clin Neurosci Res 6:237–245. 10.1016/J.CNR.2006.09.004 19169437 10.1016/j.cnr.2006.09.004 PMC2630233

[CR20] Cenini G, Lloret A, Cascella R (2020) Oxidative stress and mitochondrial damage in neurodegenerative diseases: from Molecular mechanisms to targeted therapies. Oxidative Med Cell Longev 4:20201270256. 10.1155/2020/1270256 10.1155/2020/1270256 PMC7222558 32454930

[CR21] Cerbai F, Lana D, Nosi D et al (2012) The neuron-astrocyte-microglia triad in normal brain ageing and in a model of neuroinflammation in the rat hippocampus. PLoS ONE 7(9):e45250. 10.1371/JOURNAL.PONE.0045250 23028880 10.1371/journal.pone.0045250 PMC3445467

[CR22] Cerulla N, Arcusa À, Navarro JB et al (2019) Cognitive impairment following chemotherapy for breast cancer: the impact of practice effect on results. J Clin Exp Neuropsychol 41:290–299. 10.1080/13803395.2018.1546381 30477390 10.1080/13803395.2018.1546381

[CR23] Chen C, Zhang H, Xu H et al (2019) Ginsenoside Rb1 ameliorates cisplatin-induced learning and memory impairments. J Ginseng Res 43:499–507. 10.1016/J.JGR.2017.07.009 31695559 10.1016/j.jgr.2017.07.009 PMC6823748

[CR24] Das A, Ranadive N, Kinra M et al (2020) An overview on chemotherapy-induced cognitive impairment and potential role of antidepressants. Curr Neuropharmacol 18:838–851. 10.2174/1570159X18666200221113842 32091339 10.2174/1570159X18666200221113842 PMC7569321

[CR25] De Oliveira WH, De Santana Nunes AK, De França MER et al (2016) Effects of metformin on inflammation and short-term memory in streptozotocin-induced diabetic mice. Brain Res 1644:149–160. 10.1016/J.BRAINRES.2016.05.013 27174003 10.1016/j.brainres.2016.05.013

[CR26] Dong X, Fan J, Lin D et al (2022) Captopril alleviates epilepsy and cognitive impairment by attenuation of C3-mediated inflammation and synaptic phagocytosis. J Neuroinflamm 19:226. 10.1186/S12974-022-02587-8 10.1186/s12974-022-02587-8 PMC9476304 36104755

[CR75] dos Santos NAG, Ferreira RS, dos Santos AC (2020) Overview of cisplatin-induced neurotoxicity and ototoxicity, and the protective agents. Food Chem Toxicology: Int J Published Br Industrial Biol Res Association 136:111079. 10.1016/J.FCT.2019.111079 10.1016/j.fct.2019.111079 31891754

[CR27] El-Agamy SE, Abdel-Aziz AK, Wahdan S et al (2018) Astaxanthin ameliorates Doxorubicin-Induced Cognitive Impairment (Chemobrain) in experimental rat model: impact on oxidative, inflammatory, and Apoptotic Machineries. Mol Neurobiol 55:5727–5740. 10.1007/S12035-017-0797-7 29039023 10.1007/s12035-017-0797-7

[CR28] Elmarakby AA, Sullivan JC (2012) Relationship between oxidative stress and inflammatory cytokines in diabetic nephropathy. Cardiovasc Ther 30:49–59. 10.1111/J.1755-5922.2010.00218.X 20718759 10.1111/j.1755-5922.2010.00218.x

[CR29] Fernandez HR, Varma A, Flowers SA, Rebeck GW (2020) Cancer Chemotherapy related cognitive impairment and the impact of the Alzheimer’s disease risk factor APOE. Cancers 12:1–25. 10.3390/CANCERS12123842 10.3390/cancers12123842 PMC7766535 33352780

[CR30] Ferrari-Souza JP, Ferreira PCL, Bellaver B et al (2022) Astrocyte biomarker signatures of amyloid-β and tau pathologies in Alzheimer’s disease. Mol Psychiatry 27:4781–4789. 10.1038/S41380-022-01716-2 35948658 10.1038/s41380-022-01716-2 PMC9734046

[CR31] Fouad AA, Al-Mulhim AS, Jresat I, Morsy MA (2013) Protective effects of captopril in diabetic rats exposed to ischemia/reperfusion renal injury. J Pharm Pharmacol 65:243–252. 10.1111/J.2042-7158.2012.01585.X 23278692 10.1111/j.2042-7158.2012.01585.x

[CR32] Fulco BCW, Jung JTK, Chagas PM et al (2018) Pattern differences between newborn and adult rats in cisplatin-induced hepatorenal toxicity. Chemico-Biol Interact 294:65–73. 10.1016/J.CBI.2018.08.011 10.1016/j.cbi.2018.08.011 30125553

[CR33] Gan Z, Huang D, Jiang J et al (2018) Captopril alleviates hypertension-induced renal damage, inflammation, and NF-κB activation. Brazilian J Med Biol Res = Revista brasileira de pesquisas medicas e Biologicas 51(11):e7338. 10.1590/1414-431X20187338 10.1590/1414-431X20187338 PMC6125835 30183974

[CR34] Gao Y, O’Caoimh R, Healy L et al (2013) Effects of centrally acting ACE inhibitors on the rate of cognitive decline in dementia. BMJ Open 3:e002881. 10.1136/BMJOPEN-2013-002881 23887090 10.1136/bmjopen-2013-002881 PMC3703568

[CR35] Ghosh S (2019) Cisplatin: the first metal based anticancer drug. Bioorg Chem 88:102925. 10.1016/J.BIOORG.2019.102925 31003078 10.1016/j.bioorg.2019.102925

[CR36] Gupta P, Makkar TK, Goel L, Pahuja M (2022) Role of inflammation and oxidative stress in chemotherapy-induced neurotoxicity. Immunol Res 70:725–741. 10.1007/S12026-022-09307-7 35859244 10.1007/s12026-022-09307-7

[CR37] Hannon E, Dempster EL, Davies JP et al (2024) Quantifying the proportion of different cell types in the human cortex using DNA methylation profiles. BMC Biol 22(1):17. 10.1186/S12915-024-01827-Y 38273288 10.1186/s12915-024-01827-y PMC10809680

[CR38] Hassan MM, Wahdan SA, El-Naga RN et al (2024) Ondansetron attenuates cisplatin-induced behavioral and cognitive impairment through downregulation of NOD-like receptor inflammasome pathway. Toxicol Appl Pharmcol 485:116875. 10.1016/J.TAAP.2024.116875 10.1016/j.taap.2024.116875 38437957

[CR39] Ibrahim KM, Darwish SF, Mantawy EM, El-demerdash E (2024) Molecular mechanisms underlying cyclophosphamide-induced cognitive impairment and strategies for neuroprotection in preclinical models. Mol Cell Biochem 479(8):1873–1893. 10.1007/S11010-023-04805-0 37522975 10.1007/s11010-023-04805-0 PMC11339103

[CR40] Ishola IO, Afolayan OO, Badru WA et al (2022) Angiotensin converting enzyme inhibitor captopril prevents neuronal overexpression of amyloid-beta and alpha-synuclein in Drosophila melanogaster genetic models of neurodegenerative diseases. Nigerian J Physiological Sciences: Official Publication Physiological Soc Nigeria 37:21–28. 10.54548/NJPS.V37I1.3 10.54548/njps.v37i1.3 35947848

[CR42] Jacobs SS, Fox E, Dennie C et al (2005) Plasma and cerebrospinal fluid pharmacokinetics of intravenous oxaliplatin, cisplatin, and carboplatin in nonhuman primates. Clin cancer Research: Official J Am Association Cancer Res 11:1669–1674. 10.1158/1078-0432.CCR-04-1807 10.1158/1078-0432.CCR-04-1807 15746072

[CR41] Jacobs S, McCully CL, Murphy RF et al (2010) Extracellular fluid concentrations of cisplatin, carboplatin, and oxaliplatin in brain, muscle, and blood measured using microdialysis in nonhuman primates. Cancer Chemother Pharmacol 65:817–824. 10.1007/S00280-009-1085-7 19662415 10.1007/s00280-009-1085-7 PMC6936607

[CR43] Jaiswara PK, Shukla SK (2023) Chemotherapy-mediated neuronal aberration. Pharmaceuticals (Basel Switzerland) 16(8):1165. 10.3390/PH16081165 37631080 10.3390/ph16081165 PMC10459787

[CR44] Janelsins MC, Kesler SR, Ahles TA, Morrow GR (2014) Prevalence, mechanisms, and management of cancer-related cognitive impairment. Int Rev Psychiatry (Abingdon) 26:102–113. 10.3109/09540261.2013.864260 10.3109/09540261.2013.864260 PMC4084673 24716504

[CR45] Jangra A, Kwatra M, Singh T et al (2016) Edaravone alleviates cisplatin-induced neurobehavioral deficits via modulation of oxidative stress and inflammatory mediators in the rat hippocampus. Eur J Pharmacol 791:51–61. 10.1016/J.EJPHAR.2016.08.003 27492363 10.1016/j.ejphar.2016.08.003

[CR46] John T, Lomeli N, Bota DA (2017) Systemic cisplatin exposure during infancy and adolescence causes impaired cognitive function in adulthood. Behav Brain Res 319:200–206. 10.1016/J.BBR.2016.11.013 27851909 10.1016/j.bbr.2016.11.013 PMC5332150

[CR47] Kalia K, Narula GD, Kannan GM, Flora SJS (2007) Effects of combined administration of captopril and DMSA on arsenite induced oxidative stress and blood and tissue arsenic concentration in rats. Comp Biochem Physiol Toxicol Pharmacology: CBP 144:372–379. 10.1016/J.CBPC.2006.11.001 10.1016/j.cbpc.2006.11.001 17188940

[CR48] Karimani A, Mamashkhani Y, Jafari AM et al (2018) Captopril attenuates Diazinon-Induced oxidative stress: a subchronic study in rats. Iran J Med Sci 43:514–522 30214104 PMC6123549

[CR49] Kim KY, Shin KY, Chang KA (2023) GFAP as a potential biomarker for Alzheimer’s Disease: a systematic review and Meta-analysis. Cells 12(9):1309. 10.3390/CELLS12091309 37174709 10.3390/cells12091309 PMC10177296

[CR50] Kimelberg HK, Nedergaard M (2010) Functions of astrocytes and their potential as therapeutic targets. Neurotherapeutics: J Am Soc Experimental Neurother 7:338–353. 10.1016/J.NURT.2010.07.006 10.1016/j.nurt.2010.07.006 PMC2982258 20880499

[CR51] Kleih M, Böpple K, Dong M et al (2019) Direct impact of cisplatin on mitochondria induces ROS production that dictates cell fate of ovarian cancer cells. Cell Death Dis 10(11):851. 10.1038/S41419-019-2081-4 31699970 10.1038/s41419-019-2081-4 PMC6838053

[CR52] Kumburovic I, Selakovic D, Juric T et al (2019) Antioxidant effects of Satureja hortensis L. Attenuate the Anxiogenic Effect of Cisplatin in rats. Oxidative medicine and cellular longevity 2019:8307196. 10.1155/2019/8307196 10.1155/2019/8307196 PMC6701305 31467638

[CR53] Lawrence T (2009) The nuclear factor NF-kappaB pathway in inflammation. Cold Spring Harb Perspect Biol 1(6):a001651. 10.1101/CSHPERSPECT.A001651 20457564 10.1101/cshperspect.a001651 PMC2882124

[CR54] Lingappan K (2018) NF-κB in oxidative stress. Curr Opin Toxicol 7:81–86. 10.1016/j.cotox.2017.11.002 29862377 10.1016/j.cotox.2017.11.002 PMC5978768

[CR56] Liu Y, Sun JD, Song LK et al (2015) Environment-contact administration of rotenone: a new rodent model of Parkinson’s disease. Behav Brain Res 294:149–161. 10.1016/J.BBR.2015.07.058 26239001 10.1016/j.bbr.2015.07.058

[CR55] Liu T, Zhang L, Joo D, Sun SC (2017) NF-κB signaling in inflammation. Signal Transduct Target Therapy 2:17023. 10.1038/SIGTRANS.2017.23 10.1038/sigtrans.2017.23 PMC5661633 29158945

[CR57] Lomeli N, Di K, Czerniawski J et al (2017) Cisplatin-induced mitochondrial dysfunction is associated with impaired cognitive function in rats. Free Radic Biol Med 102:274–286. 10.1016/J.FREERADBIOMED.2016.11.046 27908784 10.1016/j.freeradbiomed.2016.11.046 PMC5308450

[CR58] Lomeli N, Lepe J, Gupta K, Bota DA (2021) Cognitive complications of cancer and cancer-related treatments - novel paradigms. Neurosci Lett 749:135720. 10.1016/J.NEULET.2021.135720 33582187 10.1016/j.neulet.2021.135720 PMC8423125

[CR59] Mahmoud AMA, Mantawy EM, Wahdan SA et al (2023) Vildagliptin restores cognitive function and mitigates hippocampal neuronal apoptosis in cisplatin-induced chemo-brain: imperative roles of AMPK/Akt/CREB/ BDNF signaling cascades. Biomed Pharmacotherapy = Biomedecine Pharmacotherapie 159:114238–114238. 10.1016/J.BIOPHA.2023.114238 10.1016/j.biopha.2023.114238 36640673

[CR60] Messerli FH, Bangalore S, Bavishi C, Rimoldi SF (2018) Angiotensin-converting enzyme inhibitors in hypertension: to use or not to use? J Am Coll Cardiol 71:1474–1482. 10.1016/J.JACC.2018.01.058 29598869 10.1016/j.jacc.2018.01.058

[CR61] Miedel CJ, Patton JM, Miedel AN et al (2017) Assessment of spontaneous alternation, Novel Object Recognition and limb clasping in transgenic mouse models of Amyloid-β and Tau Neuropathology. J Visualized Experiments: JoVE 2017(123):55523. 10.3791/55523 10.3791/55523 PMC5608159 28605382

[CR62] Miners JS, Ashby E, Baig S et al (2009) Angiotensin-converting enzyme levels and activity in Alzheimer’s disease: differences in brain and CSF ACE and association with ACE1 genotypes. Am J Translational Res 1:163–177 PMC2776311 19956428

[CR63] Mogi M, Iwanami J, Horiuchi M (2012) Roles of brain angiotensin II in cognitive function and dementia. Int J Hypertens 2012:2012:169649. 10.1155/2012/169649 23304450 10.1155/2012/169649 PMC3529904

[CR64] Mostafa F, Mantawy EM, Azab SS, El-Demerdash E (2019) The angiotensin converting enzyme inhibitor captopril attenuates testosterone-induced benign prostatic hyperplasia in rats; a mechanistic approach. Eur J Pharmacol 865:172729. 10.1016/J.EJPHAR.2019.172729 31605676 10.1016/j.ejphar.2019.172729

[CR65] Mounier NM, Abdel-Maged AES, Wahdan SA et al (2020) Chemotherapy-induced cognitive impairment (CICI): an overview of etiology and pathogenesis. Life Sci 258:118071. 10.1016/J.LFS.2020.118071 32673664 10.1016/j.lfs.2020.118071

[CR66] Muñoz A, Rey P, Guerra MJ et al (2006) Reduction of dopaminergic degeneration and oxidative stress by inhibition of angiotensin converting enzyme in a MPTP model of parkinsonism. Neuropharmacology 51:112–120. 10.1016/J.NEUROPHARM.2006.03.004 16678218 10.1016/j.neuropharm.2006.03.004

[CR67] Országhová Z, Mego M, Chovanec M (2021) Long-term cognitive dysfunction in Cancer survivors. Front Mol Biosci 8:770413. 10.3389/FMOLB.2021.770413 34970595 10.3389/fmolb.2021.770413 PMC8713760

[CR68] Owoeye O, Adedara IA, Farombi EO (2018) Pretreatment with taurine prevented brain injury and exploratory behaviour associated with administration of anticancer drug cisplatin in rats. Biomed Pharmacotherapy = Biomedecine Pharmacotherapie 102:375–384. 10.1016/J.BIOPHA.2018.03.051 29571023 10.1016/j.biopha.2018.03.051

[CR69] Piccolini VM, Esposito A, Dal Bo V et al (2015) Cerebellum neurotransmission during postnatal development: [Pt(o,O’-acac)(γ-acac)(DMS)] vs cisplatin and neurotoxicity. Int J Dev Neuroscience: Official J Int Soc Dev Neurosci 40:24–34. 10.1016/J.IJDEVNEU.2014.10.006 10.1016/j.ijdevneu.2014.10.006 25450526

[CR70] Qi L, Luo Q, Zhang Y et al (2019) Advances in Toxicological Research of the Anticancer Drug Cisplatin. Chem Res Toxicol 32:1469–1486. 10.1021/ACS.CHEMRESTOX.9B00204 31353895 10.1021/acs.chemrestox.9b00204

[CR71] Rendeiro C, Sheriff A, Bhattacharya TK et al (2016) Long-lasting impairments in adult neurogenesis, spatial learning and memory from a standard chemotherapy regimen used to treat breast cancer. Behav Brain Res 315:10–22. 10.1016/J.BBR.2016.07.043 27478140 10.1016/j.bbr.2016.07.043

[CR72] Rodríguez-Giraldo M, González-Reyes RE, Ramírez-Guerrero S et al (2022) Astrocytes as a therapeutic target in Alzheimer’s Disease-Comprehensive Review and recent developments. Int J Mol Sci 23(21):13630. 10.3390/IJMS232113630 36362415 10.3390/ijms232113630 PMC9654484

[CR73] Romani AMP (2022) Cisplatin in cancer treatment. Biochem Pharmacol 206:115323. 10.1016/J.BCP.2022.115323 36368406 10.1016/j.bcp.2022.115323

[CR74] Rygiel K (2016) Can angiotensin-converting enzyme inhibitors impact cognitive decline in early stages of Alzheimer’s disease? An overview of research evidence in the elderly patient population. J Postgrad Med 62:242–248. 10.4103/0022-3859.188553 27763482 10.4103/0022-3859.188553 PMC5105210

[CR76] Santos JC, Pyter LM (2018) Neuroimmunology of behavioral comorbidities Associated with Cancer and Cancer treatments. Front Immunol 9:1195. 10.3389/FIMMU.2018.01195 29930550 10.3389/fimmu.2018.01195 PMC6001368

[CR77] Saral S, Topçu A, Alkanat M et al (2021) Apelin-13 activates the hippocampal BDNF/TrkB signaling pathway and suppresses neuroinflammation in male rats with cisplatin-induced cognitive dysfunction. Behav Brain Res 408:113290. 10.1016/J.BBR.2021.113290 33845103 10.1016/j.bbr.2021.113290

[CR78] Simpson DSA, Oliver PL (2020) ROS Generation in Microglia: understanding oxidative stress and inflammation in neurodegenerative disease. Antioxidants 9:1–27. 10.3390/ANTIOX9080743 10.3390/antiox9080743 PMC7463655 32823544

[CR79] Sofroniew MV (2020) Astrocyte reactivity: subtypes, states and functions in CNS innate immunity. Trends Immunol 41:758–770. 10.1016/J.IT.2020.07.004 32819810 10.1016/j.it.2020.07.004 PMC7484257

[CR80] Solleiro-Villavicencio H, Rivas-Arancibia S (2018) Effect of chronic oxidative stress on neuroinflammatory response mediated by CD4 + T cells in neurodegenerative diseases. Front Cell Neurosci 12:114. 10.3389/FNCEL.2018.00114 29755324 10.3389/fncel.2018.00114 PMC5934485

[CR81] Sonsalla PK, Coleman C, Wong LY et al (2013) The angiotensin converting enzyme inhibitor captopril protects nigrostriatal dopamine neurons in animal models of parkinsonism. Exp Neurol 250:376–383. 10.1016/J.EXPNEUROL.2013.10.014 24184050 10.1016/j.expneurol.2013.10.014 PMC3889207

[CR82] Takuathung MN, Sakuludomkan W, Khatsri R et al (2022) Adverse effects of Angiotensin-converting enzyme inhibitors in humans: a systematic review and Meta-analysis of 378 randomized controlled trials. Int J Environ Res Public Health 19(14):8373. 10.3390/IJERPH19148373 35886227 10.3390/ijerph19148373 PMC9324875

[CR83] Tao MX, Xue X, Gao L et al (2018) Involvement of angiotensin-(1–7) in the neuroprotection of captopril against focal cerebral ischemia. Neurosci Lett 687:16–21. 10.1016/J.NEULET.2018.09.024 30219484 10.1016/j.neulet.2018.09.024

[CR84] Taskiran AS, Avci O (2021) Effect of captopril, an angiotensin-converting enzyme inhibitor, on morphine analgesia and tolerance in rats, and elucidating the inflammation and endoplasmic reticulum stress pathway in this effect. Neurosci Lett 741:135504. 10.1016/J.NEULET.2020.135504 33197521 10.1016/j.neulet.2020.135504

[CR85] Troy L, McFarland K, Littman-Power S, Kelly BJ, Walpole ET, Wyld D, Thomson D (2000) Cisplatin-based therapy: a neurological and neuropsychological review. Psycho-oncology 9(1):29–39. 10.1002/(SICI)1099-1611(200001/02)9:1%3C;29::AID-PON428%3E;3.0.CO;2-Z 10668057 10.1002/(sici)1099-1611(200001/02)9:1<29::aid-pon428>3.0.co;2-z

[CR86] Uchiyama M, Mihara M (1978) Determination of malonaldehyde precursor in tissues by thiobarbituric acid test. Anal Biochem 86:271–278. 10.1016/0003-2697(78)90342-1 655387 10.1016/0003-2697(78)90342-1

[CR87] Van Dorst DCH, Dobbin SJH, Neves KB et al (2021) Hypertension and Prohypertensive Antineoplastic therapies in Cancer patients. Circul Res 128:1040–1061. 10.1161/CIRCRESAHA.121.318051 10.1161/CIRCRESAHA.121.318051 PMC8011349 33793337

[CR88] von Bartheld CS, Bahney J, Herculano-Houzel S (2016) The search for true numbers of neurons and glial cells in the human brain: a review of 150 years of cell counting. J Comp Neurol 524:3865–3895. 10.1002/CNE.24040 27187682 10.1002/cne.24040 PMC5063692

[CR89] Walker KA, Power MC, Gottesman RF (2017) Defining the relationship between hypertension, cognitive decline, and dementia: a review. Curr Hypertens Rep 19:(3):24. 10.1007/S11906-017-0724-3 28299725 10.1007/s11906-017-0724-3 PMC6164165

[CR90] Wardill HR, Mander KA, Van Sebille YZA et al (2016) Cytokine-mediated blood brain barrier disruption as a conduit for cancer/chemotherapy-associated neurotoxicity and cognitive dysfunction. Int J Cancer 139:2635–2645. 10.1002/IJC.30252 27367824 10.1002/ijc.30252

[CR91] Yoo KH, Tang JJ, Rashid MA et al (2021) Nicotinamide mononucleotide prevents cisplatin-induced cognitive impairments. Cancer Res 81:3727–3737. 10.1158/0008-5472.CAN-20-3290 33771896 10.1158/0008-5472.CAN-20-3290 PMC8277702

[CR92] Yu H, Lin L, Zhang Z et al (2020) Targeting NF-κB pathway for the therapy of diseases: mechanism and clinical study. Signal Transduct Target Therapy 5(1):209. 10.1038/S41392-020-00312-6 10.1038/s41392-020-00312-6 PMC7506548 32958760

[CR93] Zhang S, Wu M, Peng C et al (2017) GFAP expression in injured astrocytes in rats. Experimental Therapeutic Med 14:1905–1908. 10.3892/ETM.2017.4760 10.3892/etm.2017.4760 PMC5609138 28962102

[CR94] Zhou W, Kavelaars A, Heijnen CJ (2016) Metformin prevents Cisplatin-Induced Cognitive impairment and brain damage in mice. PLoS ONE 11(3):e0151890. 10.1371/JOURNAL.PONE.0151890 27018597 10.1371/journal.pone.0151890 PMC4809545

